# The Protective Effect of* Chrysanthemum indicum* Extract against Ankylosing Spondylitis in Mouse Models

**DOI:** 10.1155/2017/8206281

**Published:** 2017-02-02

**Authors:** Mei Dong, Dongsheng Yu, Veeramuthu Duraipandiyan, Naif Abdullah Al-Dhabi

**Affiliations:** ^1^Infectious Immune Department of Rheumatism, Tianjin Hospital, Jiefangnan Road 406, Tianjin 300211, China; ^2^Department of Rehabilitation Medicine, Tianjin Medical University, Tianjin 300052, China; ^3^Department of Botany and Microbiology, Addiriyah Chair for Environmental Studies, College of Science, King Saud University, P.O. Box 2455, Riyadh 11451, Saudi Arabia

## Abstract

In traditional Chinese and Korean homeopathic medicine,* Chrysanthemum indicum* Linné (Asteraceae) is a time-honored herb, prescribed for the resolution of symptoms associated with inflammatory and hypertensive conditions as well as those affecting the lungs and its associated structures. The goal of this work is to investigate the defensive role of* Chrysanthemum indicum* extract in fighting ankylosing spondylitis (AS) using mouse models, through which the manifestation and extent of the disease progression were measured with quantitative analysis of the intervertebral joints. Markers of inflammation as well as oxidative stress were also analysed. Western blot was used to quantify the levels of Nuclear Factor-*κ*B (NF-*κ*B) p65, Dickkopf-1 (DKK-1), and sclerostin (SOST). Consequently, the findings of this experiment demonstrated that AS in mice that were given* Chrysanthemum indicum* extract had lower level of TNF-*α*, IL-1*β*, and IL-6 (*P* < 0.05) and increased level of catalase (CAT), glutathione peroxidase (GSH-PX), and superoxide dismutase (SOD) (*P* < 0.05). The results also revealed that* Chrysanthemum indicum* supplemented with diet contributed to a decrease in Nuclear Factor-*κ*B (NF-*κ*B) p65 protein expression (*P* < 0.05) and higher levels of DKK-1 and SOST proteins (*P* < 0.05). Therefore, we concluded that the beneficial role of* Chrysanthemum indicum* in AS is manifested through downregulating oxidative stress, inhibiting inflammatory mediators and NF-*κ*B, and increasing DKK-1 and SOST levels.

## 1. Introduction

AS can be described as an autoimmune disease in which the disease is presented by inflammation of the sacroiliac and axial joints as well as stiffness of the spine [1]. It most commonly affects men in their 30s under the guise of chronic back pain [2]. The areas most affected by AS are the spinal and sacroiliac joints, with more severe cases affecting the entire spine [3]. Symptoms of AS include tenderness in the lumbar regions, radiating to the shoulders and neck which can occur with or without spasticity; other symptoms include foot pain, more often seen in the heel, pain that is progressively worse during the night, trouble sleeping as well as debilitating spasticity affecting the waist-noticeable after long periods in a supine state [4]. AS can be described as an autoimmune disease that is multifactorial but has a strong genetic susceptibility, placed under the category of spondyloarthorpathies.

Elevated concentrations of reactive oxygen species (ROS) and proinflammatory cytokines and are implicated in this disease, with its most devastating consequences being the eventual transition from inflammation to the production of excess bone. The way in which this is thought to occur is still unknown, but it is stipulated that an inflammatory cascade is stimulated at the interface of tendon/ligaments and bone, causing the creation of bony projections known as syndesmophytes. With time and the progressive worsening of the disease, these bony projections will fuse and resultant ankyloses will occur [5, 6]. Although the precise way in which AS occurs is largely unknown, proinflammatory mediators are strongly implicated in the initiation and progression of this disease. Their role in stimulating osteoclasts as well as other inflammatory cells to further produce TNF-*α* and IL-6 has been well established [3, 7].

It is considered that the most reliable way to extrapolate the findings of such research to humans are to use animal models. AS has been investigated in a variety of models,including that of mice, in which collagen induced arthritis has been studied [8] as well as proteoglycan-induced arthritis (PGIA). Interestingly, the role of ROS generated within certain cells of the human body has received more and more attention. ROS as well as reactive nitrogen species are what is known as free radicals, produced during normal metabolic reactions within our bodies. However, very large concentrations and uncontrolled elevated amounts of these species are associated with oxidative stress [9].

CAT, SOD, and GSH-PX are important antioxidant enzymes, which have a scavenging role in superoxide radical removal in cells. The significance of these processes is that they help to lower the toxic nature of free radicals, thereby limiting potential harm to our bodies [10]. As more research and understanding is needed in the field of AS and the knowledge we have at present in relation to the way in which AS is initiated and develops is poor, the available treatment methods are limited as a consequence. Newer studies in animal and human models have discovered the Wingless (Wnt) pathway, which is considered to be crucial in bone production.

The use of the herb* Chrysanthemum indicum* Linné (Compositae) has been well established in Korean, Chinese, and Japanese medicine for hundreds of years, where it is used in the treatment of autoimmune diseases. Its applications include diseases which cause inflammation and elevated blood pressure as well as those that target the respiratory apparatus of the human body [11, 12]. Numerous experiments have concluded that* C. indicum* possess particularly potent effects on bacteria and viruses as well as having antioxidant, anti-inflammatory, and immunomodulatory properties [13, 14]. The phytochemical picture of this herb demonstrates the presence of constituents such as flavonoids, terpenoids, and phenol [15], which have been found and isolated in this herb. More specifically, evidence suggests that these components have the capacity to inhibit nitric oxide (NO) [11, 15].* C. indicum* also contains butanol, which plays an important role in inhibiting auricle edema in mouse model [13]. However, more researches are still needed to pinpoint the exact mechanisms by which* Chrysanthemum indicum* affects the molecular mechanisms seen in AS. In this research, we evaluated the effects of* Chrysanthemum indicum* extract on the severity of AS, antioxidative enzymes, proinflammatory cytokines production, NF-*κ*B, and Wnt pathway.

## 2. Materials and Methods

### 2.1. Extraction and Preparation of* Chrysanthemum indicum* Extract

In order to conduct this experience, the bud of* Chrysanthemum indicum* was required, sourced from a nearby market and scrutinized for authenticity based on their micro- and macroscopic qualities. The parts we required from* Chrysanthemum indicum* were then obtained by treating it with 70% ethanol over a reflux process of two hours. The residual parts were then concentrated using high pressure and filtered, lyophilized, and dissolved in 10% dimethyl sulfoxide (DMSO). Then the samples were filtered by using a 0.2-*μ*m syringe filter and lyophilized.

### 2.2. Creation of the AS Mouse Model

The animal models in this experiment were treated in line with the guidelines as set by Tianjin Hospital (Tianjin, China). BALB/c mice sourced from the Tianjin Laboratory Animal Center (Tianjin, China). Forty mice were allotted to four treatment groups with a completely randomized design (*n* = 10 per group): (i) control group (CTRL), which contained healthy control mice and received a basal diet [16]; (ii) control ankylosing spondylitis group (CTRL-AS), which contained AS mice and received a basal diet; (iii)* Chrysanthemum indicum* extract group (CIE), which contained normal healthy mice and received a basal diet and 30 mg/kg* Chrysanthemum indicum* extract; (iv)* Chrysanthemum indicum* extract ankylosing spondylitis group (CIE-AS), which included AS mice and received a basal diet and 30 mg/kg* Chrysanthemum indicum* extract. In control-AS group and CIE-AS group, three-month-old mice was injected with 2 mg human proteoglycan extract dissolved in 2 mg dimethyldioctadecylammonium (DDA) for 4 times at two-week intervals, in order to produce symptoms of spondylitis [17]. After the second injection, we noticed symptoms in line with peripheral arthritis, with the mice demonstrating signs of axial skeleton ankyloses [17].

The mice were then marked for signs and symptoms of arthritis [18]: 0 (no symptoms), 1 (redness and swelling in one toe), 2 (redness and swelling in more than one toe), 3 (toe stiffness), and 4 (deformity or ankle involvement). A total score was obtained by adding up the value of all the final scores obtained for each toe. At the final stage of the experiment, the mice were examined using histological analysis and blood was drawn from the retroorbital plexus. Small amounts of flash-frozen plasma were created and stored at a temperature of −80°C.

### 2.3. Histological Scoring

Tissue that was taken from the vertebra was prepared in the laboratory and stained by the hematoxylin and eosin (H & E) method. These were allocated a number from 0 to 4 with a specific numbering system [19]. The scoring, which assessed the degree to which the spine was affected, took place in the following way: (1) enthesitis, presence of cellular components that initiate inflammation, around the intervertebral disc (IVD) with permeation of the annulus fibrosus; (2) less than 50% of the IVD being affected; (3) almost all of the IVD being affected (greater than 50%); (4) bony fusion/fusion of the cartilage. At least 18–22 IVDs (from cervical portions of the lumbar area) of every control and injected mice were marked. Finally, the spondylitis score in every mouse was calculated by dividing all the IVD scores, by the number of IVDs which was looked at microscopically. The criterion for inclusion was that only tissue sections that were treated and fixated properly could be considered for the grading, that is, tissue sections with a clear nucleus as well as a fibrotic annulus. The average grade was taken for each sample in order to allow us to detect similarities and changes.

### 2.4. The Analysis of Cytokines and Antioxidative Enzyme Activities

The TNF-*α*, IL-1*β*, and IL-6 in the serum were quantified using ELISA kits (Jiancheng Bioengineering Institute, China). MDA, CAT, SOD, and GSH-PX activities were also measured using ELISA and interpreted using the standard curve (Jiancheng Bioengineering Institute, China). The optical densities were measured at 405 nm with a Bio-Rad microplate reader (Bio-Rad Laboratories, USA).

### 2.5. Western Blot of NF-*κ*B p65, Dickkopf-1 (DKK-1), and Sclerostin (SOST) in AS Mouse Model

When the study was coming to a close, 10 mg of AS tissue was extracted and preserved with 100 *μ*L tissue lysis buffer (Jiancheng Bioengineering Institute, Nanjing, China) for half an hour using ice. A BCA kit (Bio-Rad Laboratories, USA) was utilized in order to quantify the amount of protein present. The same quantity of protein (50 *μ*g) was measured and 12% SDS-polyacrylamide gel was placed onto a polyvinylidene fluoride membrane (Bio-Rad Laboratories, USA), blocked with 5% nonfat milk and primary antibodies and then incubated at 4°C. After this, it was incubated alongside an anti-rabbit secondary antibody for 30 min at 37°C, created with the help of an EasyBlot ECL kit (Jiancheng Bioengineering Institute, China) in line with the instructions given by the producer. Band intensities were measured with Quantity One (V4.4.0, Bio-Rad Laboratories, USA).

### 2.6. Statistical Analysis

Data were presented as the mean ± standard deviation. The analyses were performed using SPSS (V22.0, IBM, USA). The Student's* t*-test was the method of choice to determine statistical significance with *P* < 0.05 demonstrating a statistical significance.

## 3. Results

### 3.1. Effects of Dietary* Chrysanthemum indicum* Extract on the Process and Severity of AS

**Figure 1:**
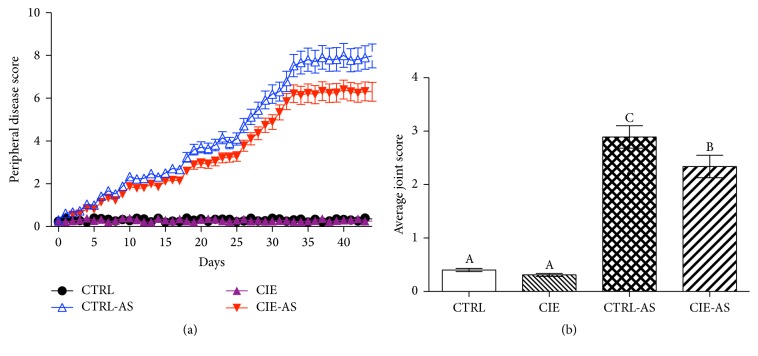
Impact of dietary* Chrysanthemum indicum* extract on (a) peripheral disease progression and (b) average joints score. Different superscripts shown on the data indicate being statistically different from each other (*P* < 0.05; *n* = 10).

In CTRL-AS and CIE-AS groups, peripheral arthritis can be evaluated by monitoring the paw swelling and stiffness of rear leg joints. Dietary* Chrysanthemum indicum* extract delayed (*P* < 0.05) the progression of peripheral disease (Figure 1(a)) in CIE-AS group in comparison to that in CTRL-AS group. Score of stained intervertebral joints showed that dietary* Chrysanthemum indicum* extract alleviated (*P* < 0.05) disease severity in the CIS-AS mice compared with the CTRL-AS mice (Figure 1(b)).

### 3.2. Effect of* Chrysanthemum indicum* Extract on Inflammatory Cytokines

**Figure 2:**
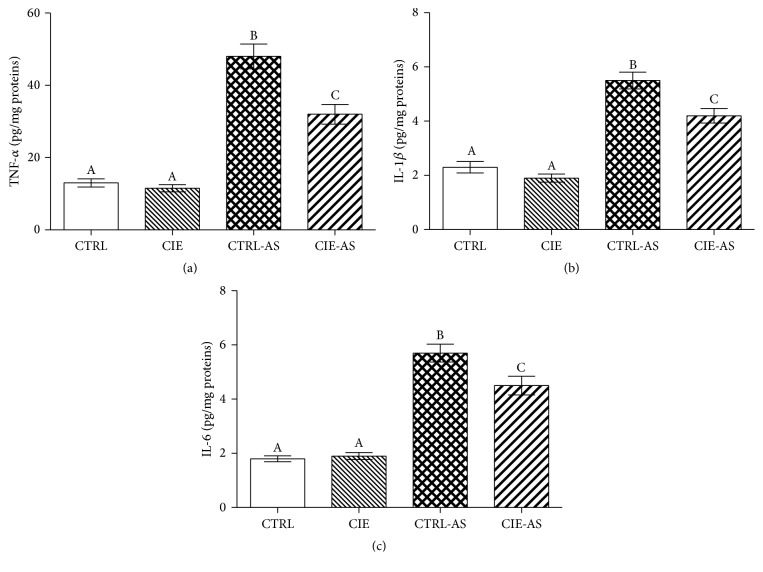
The effect of* Chrysanthemum indicum* extract on (a) TNF-*α*, (b) IL-1*β*, and (c) IL-6 in mice. Different superscripts shown on the data indicate being statistically different from each other (*P* < 0.05; *n* = 10).

In order to elucidate the precise beneficial role that* Chrysanthemum indicum* has in the animal models used in this experiment, a quantitative measure of the TNF-*α*, IL-1*β*, and IL-6 levels were obtained.  Figure 2 showed that TNF-*α*, IL-1*β*, and IL-6 levels were higher (*P* < 0.05) in the CTRL-AS and CIE-AS groups in comparison to the CTRL and CIE group. Administration with* Chrysanthemum indicum* extract resulted in the noticeable decrease in TNF-*α*, IL-1*β*, and IL-6 levels (*P* < 0.05) in the CIE-AS mice in comparison to those of the CTRL-AS mice.

### 3.3. Effect of* Chrysanthemum indicum* Extract on Levels of MDA and Antioxidative Enzymes

**Figure 3:**
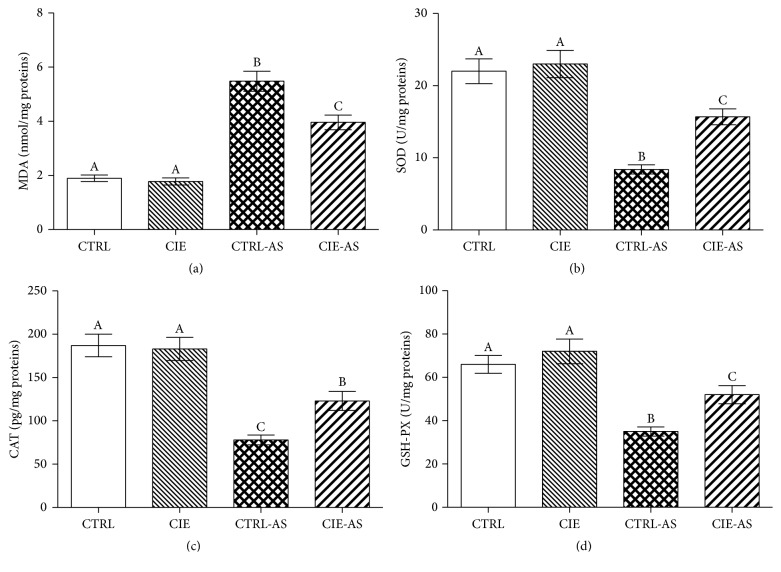
Effect of* Chrysanthemum indicum* extract on MDA and antioxidative enzymes. Dietary* Chrysanthemum indicum* extract affects concentrations of (a) MDA, (b) SOD, (c) CAT, and (d) GSH-PX. Different superscripts shown on the data indicate being statistically different from each other (*P* < 0.05; *n* = 10).

In order to demonstrate the beneficial role of* Chrysanthemum indicum* extract in reducing or eliminating oxidative stress in the mice models, the levels of MDA and antioxidative enzyme levels were measured (Figure 3). The results were such that the MDA levels were higher in the CTRL-AS and CIE-AS groups (*P* < 0.05) in comparison with the CTRL and CIE groups, whereas the SOD, CAT, and GSH-PX levels were lower (*P* < 0.05) in the CTRL-AS and CIE-AS groups (*P* < 0.05) in comparison with the CTRL and CIE groups. Following treatment with* Chrysanthemum indicum* extract, the MDA level decreased (*P* < 0.05) while SOD, CAT, and GSH-PX levels increased (*P* < 0.05) in CIE-AS group in comparison with the CTRL-AS group.

### 3.4. Effect of* Chrysanthemum indicum* Extract on NF-*κ*B p65 Unit Protein, SOST, and DKK-1 Levels

**Figure 4:**
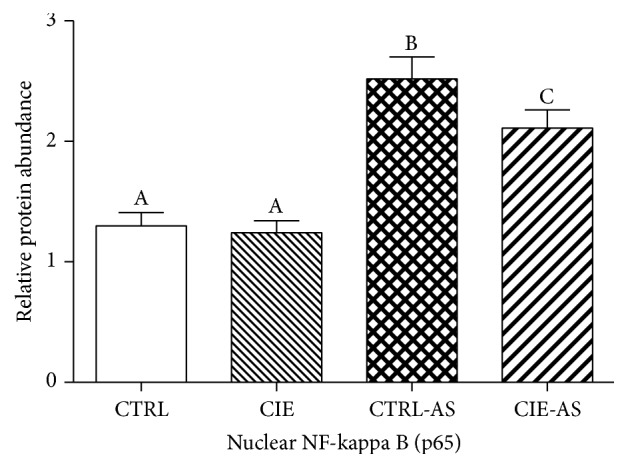
The effect of* Chrysanthemum indicum* extract on NF-*κ*B p65 protein expression in AS mouse model. Different superscripts shown on the data indicate being statistically different from each other (*P* < 0.05; *n* = 10).

To find out the beneficial role of* Chrysanthemum indicum* extract on NF-*κ*B pathway, a western blot assay analysis was carried out. The results were such that the levels of NF-*κ*B p65 were higher (*P* < 0.05) in the CTRL-AS and CIE-AS mouse groups than that in the CTRL and CIE mouse groups (Figure 4). In comparison to the CTRL-AS group, administration of the* Chrysanthemum indicum* extract caused a decrease (*P* < 0.05) in NF-*κ*B p65 protein in CIE-AS group.

**Figure 5:**
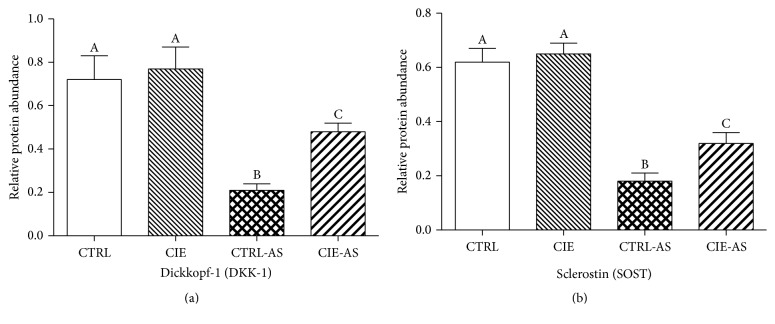
Effects of* Chrysanthemum indicum* extract on (a) SOST and (b) DKK-1 protein expression in mouse models. Different superscripts shown on the data indicate being statistically different from each other (*P* < 0.05; *n* = 10).

In order to evaluate the effects of* Chrysanthemum indicum* on Wnt pathway, the levels of SOST and DKK-1 which are inhibitors of Wnt pathway were measured (Figure 5). The results revealed that the SOST and DKK-1 protein levels were lower (*P* < 0.05) in AS mice compared with the normal mice, whereas administration of* Chrysanthemum indicum* caused an increase (*P* < 0.05) in SOST and DKK-1 level in CIE-AS mice in comparison to CTRL-AS mice (*P* < 0.05).

## 4. Discussion

AS can be described as a recurrent, chronically occurring disease of autoimmune and inflammatory nature, in which rheumatoid factor is a significant component [20]. Inflammation is localized to the vertebral and sacral joints causing a more axial presentation of the disease. Ankyloses is caused by the multiplication and growth of mesenchymal cells as well as the aggregation of a proteoglycan rich matrix full of collagen. The mineralization of this matrix subsequently leads to ankylosis. The focus of the lesion is at the origins of ligament insertions to the joint capsule. Here the inflammatory mediators act to slowly but progressively destroy the bone tissue. The resultant deficiencies are replaced by connective tissue which consists of lymph and plasma cells. The cancellous bone undergoes a form of remodeling whereby the bones surface which are worn away is filled with connective tissue, lymph, and plasma cells [21].

In individuals affected by AS, cellular components such as neutrophils are stimulated to produce ROS, which play a significant role in causing oxidative stress [10]. This is due to the fact that myeloperoxidase activity is increased, causing oxidation and peroxidation of protein components within the body. However, the levels of sulfhydryl are lowered, which leads us to believe that neutrophils play a significant part in the etiology of AS [22]. Blood samples taken from individuals with clinically evident AS demonstrated increased MDA and catalase activity, in contrast to the control group [10]. CAT activity is significantly and positively associated with the erythrocyte sedimentation rate (ESR) and concentrations of C-reactive protein. The rise in CAT levels occurs due to elevated superoxide anion levels [23]. The evidence suggests that the application of* Chrysanthemum indicum* reduces MDA levels and elevates SOD, CAT, and GSH-PX levels, as seen in CIE-AS mice in comparison to that of the AS mice. Kim et al. contest that* Chrysanthemum indicum* demonstrates a beneficial prophylactic role in experiments pertaining to Parkinson's through an antioxidative mechanism [24].

The results of this study have revealed that incorporation of* Chrysanthemum indicum* in the diet slows down the degree and pace with which the disease progresses as well as decreasing the associated involvement of the intervertebral joints. AS is categorized as a variant of a rheumatoid disease showing recurrent, chronic inflammation of joints, particularly axial ones, which may potentially affect other organs of the body as well [25]. The majority of experts in this field considers AS to be multifactorial but with a strong genetic predisposition and promoted by factors such as stress, disease, and exhaustion as well as certain external factors pertaining to the environment [26, 27]. Current evidence demonstrates that* Chrysanthemum indicum* exacerbates NF-*κ*B p65 levels as well as proinflammatory cytokine levels in the AS animal models. The findings are congruent with prior research which demonstrated that* Chrysanthemum indicum* helps to alleviate and decrease oxidative stress and inflammation [28]. Studies have also revealed that* Chrysanthemum indicum* has an inhibitory role on inflammation in particular NF-*κ*B activation in HeLa cells [29] as well as lipopolysaccharide-induced RAW 264.7 macrophages [30].

Newer studies reveal that Wnt signaling is increased in AS patients, as concentrations of bone-associated Wnt inhibitor SOST and DKK-1 are lowered in human and mouse models [17, 31]. It is well known that the Wnt pathway is a significant signaling cascade necessary for the normal production osteoblastic cells, promoting their growth and maturation [32]. In bone, DKK-1 and SOST proteins act to halt the Wnt cascade, either binding to LRP4/5/6 and stopping its connections with the Wnt-Fzd complex or connecting with Wnt proteins, blocking their roles entirely [32]. This causes us to consider the part that these proteins play in stimulating osteoblasts to grow and mature, a process commonly seen in AS [33]. Research also reveals that* Chrysanthemum indicum* increases DKK-1 and SOST concentrations, causing us to also consider that* Chrysanthemum indicum* slows down the development of the disease via dynamic alterations in the Wnt signaling pathway. The results suggested that the administration of the* Chrysanthemum indicum* has an inhibitory effect on the Wnt signaling pathway.

In conclusion,* Chrysanthemum indicum* contains a variety of beneficial components which demonstrate a beneficial role in preventing inflammation and the effects of oxidative stress. This can be attributed to the downregulation of NF-*κ*B and Wnt signaling cascades, as seen in the mouse models. However, further research and experimentation is needed to elucidate the precise beneficial role this herb has on AS.
