# Corrigendum to “The Protective Effect of* Chrysanthemum indicum* Extract against Ankylosing Spondylitis in Mouse Models”

**DOI:** 10.1155/2018/8097342

**Published:** 2018-11-01

**Authors:** Mei Dong, Dongsheng Yu, Veeramuthu Duraipandiyan, Naif Abdullah Al-Dhabi

**Affiliations:** ^1^Infectious Immune Department of Rheumatism, Tianjin Hospital, Jiefangnan Road 406, Tianjin 300211, China; ^2^Department of Rehabilitation Medicine, Tianjin Medical University, Tianjin 300052, China; ^3^Department of Botany and Microbiology, Addiriyah Chair for Environmental Studies, College of Science, King Saud University, P.O. Box 2455, Riyadh 11451, Saudi Arabia

In the article titled “The Protective Effect of* Chrysanthemum indicum* Extract against Ankylosing Spondylitis in Mouse Models” [1], there were some errors, which should be corrected as follows:

- In the Introduction section, “AS can be described as an autoimmune disease in which the disease is presented by inflammation of the sacroiliac and axial joints as well as stiffness of the spine” should be “AS is an immune-mediated disease characterized by inflammation of the sacroiliac and axial joints as well as stiffness of the spine.”
- In Histological Scoring section, “Briefly, tissues were collected and fixed in neutral buffered formalin and decalcified in 14% ethylenediaminetetraacetic acid (EDTA). Sections were stained with H&E or toluidine blue according to standard protocols.” should be added after “Tissue that was taken from the vertebra was prepared in the laboratory and stained by the hematoxylin and eosin (H & E) method.”
- In Statistical Analysis section, “The Student's t-test was the method of choice to determine statistical significance with (*P*<0.05) demonstrating a statistical significance” should be “One-way ANOVA followed by post hoc multiple comparisons (LSD method) to detect the difference level and a* P* value less than 0.05 was considered statistically different.”
- In Discussion section, “AS can be described as a recurrent, chronically occurring disease of autoimmune and inflammatory nature, in which rheumatoid factor is a significant component” should be “AS can be described as a recurrent, chronically occurring disease of autoimmune and inflammatory nature.”
- In Discussion section, “Current evidence demonstrates that* Chrysanthemum indicum* exacerbates NF-*κ*B p65 levels as well as proinflammatory cytokine levels in the AS animal models” should be “Current evidence demonstrates that* Chrysanthemum indicum* suppressed NF-*κ*B p65 levels as well as proinflammatory cytokine levels in the AS animal models.”
